# Needle Stick Injuries – Risk and Preventive Factors: A Study among Health Care Workers in Tertiary Care Hospitals in Pakistan

**DOI:** 10.5539/gjhs.v5n4p85

**Published:** 2013-04-14

**Authors:** Asad Ali Khan Afridi, Ameet Kumar, Raza Sayani

**Affiliations:** 1 Department of Community Health Aciences, Aga Khan University Hospital, Karachi, Pakistan; 2 Jinnah Postgraduate Medical Centre, Karachi, Pakistan; 3 Department of Radiology, Aga Khan University Hospital, Karachi, Pakistan

**Keywords:** needles stick injury, health care workers, hospital, Pakistan

## Abstract

**Background:**

Health care workers (HCWs) are at substantial risk of acquiring blood borne infections such as HIV, Hepatitis-B and Hepatitis-C through needle stick injuries (NSIs). This study aimed to assess the proportion of NSIs and their associated factors among HCWs and also to identify the areas in which preventive efforts might be directed to protect against this occupational hazard.

**Methodology:**

A cross-sectional study was conducted in two tertiary care hospitals of Pakistan representing both private and public health sector. A total of 497 HCWs (doctors and nurses) were interviewed using a structured questionnaire. Data was collected from January to May 2008.

**Results:**

Overall, 64% of the HCWs were exposed to at least one NSI during their career; among them 73% reported NSIs for two or more times. Factors found to be highly associated with NSIs were those practicing this occupation for more than five years (p < 0.001: OR = 5.92; 95% CI = 3.45-10.16) and working as nurse than doctor (p 0.001: OR = 2.12; 95% CI = 1.35-3.32). Having received booster dose of hepatitis B vaccine (p 0.02: OR = 1.85; 95% CI = 1.10-3.11), working in surgical specialty (p < 0.01: OR = 1.6; 95% CI = 1.09-2.51) and being a female (p 0.03: OR = 1.52; 95% CI = 1.04-2.22) were also found to be associated with NSIs. Most commonly reported reason for NSIs was injecting medicine and drawing blood (42%) followed by two-handed recapping of needle (37%). Only, 34% of study subjects were vaccinated against hepatitis B infection. Overall, HCWs had inadequate practices regarding standard precautions such as availability of gloves/protective cloths (40%) and infection control guidelines/protocols (10%) respectively in their working places.

**Conclusion:**

In addition to very high rates of NSIs, low safety practices including inadequate vaccination coverage, unavailability of infection control guidelines and other preventive facilities were reported in this study. Prevention of occupational infections among HCWs should be a priority. Formal training, by health authorities in the local area, about safe practices and availability of preventive facilities should be ensured regarding NSIs among HCWs.

## 1. Introduction

Health Care Workers (HCW) are at increased risk of getting needle stick injuries and associated blood borne infections including hepatitis B, hepatitis C and HIV (Pruss-Ustun, Rapiti, & Hutin, 2005). It is estimated that in United States approximately 385,000 needle stick and sharp-related injuries occur every year to healthcare workers in hospital settings (Centre for disease control and prevention). Globally, out of 39.5 million health-care workers (Dal Poz, Kinfu, DrÃ¤ger, Kunjumen, & Diallo, 2006), three million experience percutaneous exposure to infectious diseases each year and 40% of hepatitis B, and C and 2.5% of HIV/AIDS in HCWs are attributed to needle stick injuries (WHO, 2002; World health report, Geneva). With such high rates of transmission of blood borne diseases amongst HCW due to needle stick injuries, it is prudent to devise prevention strategies in order to limit such incidents.

While >90% of blood borne infections occurs among HCW in developing countries (WHO, 2002; World health report, Geneva; Wilburn & Eijkemans, 2004) reporting of such events is rarely done (Azap et al., 2005; Lee, 2009; Zafar et al., 2009). Furthermore, different studies have shown transmission of Hepatitis B and C from HCWs to patients (Buster, van der Eijk, de Man, & Schalm, 2004; Laurenson et al., 2007; Lot et al., 2007; Raggam, Rossmann, Salzer, Stauber, & Kessler, 2009) and approximately 70 infected health care workers have been identified in transmitting their infection to patients (Carlson & Perl, 2010). Hence significant health workers are exposed to blood borne pathogens that pose a threat to them as well as their patients, health (Chow, Rayment, Wong, Jefferys, & Suranyi, 2009).

The increased incidence of needle stick injuries in HCW is known to arise from a combination of high risk activities with low safety measures (activities including administering injections, drawing blood, recapping needles, disposing of needles, handling trash and dirty linen and transferring blood or body fluids from a syringe to a specimen container and missing the target) (Azap et al., 2005; Wilburn & Eijkemans, 2004). Such behavior not only affects the quality of care delivered by the HCW but also affects the safety and well-being of care providers. Furthermore, HCWs in high risk areas of hospital setting are potentially at an increased risk of exposure (Pruss-Ustun et al., 2005) and experience substantial anxiety and depression (Sohn, Kim, Kim, & Han, 2006), which may lead to changes in occupation and behavior. Hence, needle stick injuries not only lead to increased risk of developing infections but also affect the mental health of health care providers; as a result, strategies need to be employed to reduce the risk of sustaining such injuries.

Various studies have been conducted worldwide to assess the proportion of needle stick injuries among HCW. While several studies have been conducted to assess the proportion of HCW suffering from needle stick injuries in developed countries such as US, limited epidemiological studies regarding the risks and circumstances of needle stick and other sharp injuries among health personnel have been identified in developing countries (Moro et al., 2007) and have found significantly high proportions (Tadesse & Tadesse, 2010; Talaat et al., 2003). Different studies from Pakistan reported prevalence of NSI ranging from 45% to 94% among HCW (Siddique, Mirza, Tauqir, Anwar, & Malik, 2008; Zafar, Aslam, Nasir, Meraj, & Mehraj, 2008); however, these were conducted in a single hospital and had small sample sizes.

This high prevalence of needle stick injuries in HCW warrants to study the factors associated with it in order to device prevention strategies and decrease the chances of acquiring infections. Hence we aim to estimate the prevalence of needle stick injuries in health care workers at public and private tertiary care hospitals in Karachi, Pakistan and the factors associated with it.

## 2. Material and Methods

This was a cross sectional study conducted during the months of January to May 2008 in two tertiary care hospitals of Karachi representing public and private sectors. Study sample included a total of 515 health care workers; amongst those who took part in study, 7 were not available on the day of interview while 11 refused to take part in this study. Hence our final sample included 497 HCWs, yielding a response rate of >96%. Study participants were HCWs, working full time at tertiary care hospitals in Karachi, Pakistan for ≥ 1 year. List of all nurses and doctors from both hospitals were made and respondents were selected by simple random sampling via use of table of random numbers. Participants were included from medicine as well surgery departments. A pre-tested, structured questionnaire about basic socio demographic factors, needle stick injuries and availability of preventive facilities for such injuries was administered by trained medical students in various wards of both hospitals.

We had 2 main outcomes viz (a) prevalence of needle stick injuries and (b) availability of preventive facilities. We defined NSI as the par literal introduction into the body of healthcare worker, during the performance of their duties, of blood or other potentially hazardous material by a hollow bore needle or sharp instruments, including, but not limited to, needles, lancets, scalpels, and contaminated broken glass (Needle stick injuries, 2003)

Overall, 497 HCW were interviewed and Statistical Package for social sciences (SPSS) version 17.0 (SPSS 17.0; SPSS Inc., Chicago, IL, USA) was used to enter and analyze the data. Proportions along with 95% confidence intervals (CI) were calculated for those who reported having suffered from needle stick injuries. Chi square test and univariate analysis along with 95% CI was calculated to assess factors associated with needle stick injuries.

### 2.1 Ethical Considerations

The Research Committee of the department of Family Medicine, Aga Khan University, Karachi approved the study protocol. This was followed by approval from hospital administration. Purpose of study was explained to each participant and verbal informed consent was obtained from before getting enrolled in the study. All study participants and hospital administration were assured about the confidentiality and anonymity and efforts were made to ensure the privacy of the information.

## 3. Results

We interviewed 497 HCWs from public and private sector hospitals. Prevalence of NSIs was around 64%. Majority of the study participants were females (64.2%) and practicing in medical units (62%). Overall, 29.6% HCWs were working for more than five years while there was slight preponderance of nurses (52.7%) compared to doctors (47.3%) (Table 1).

**Table 1:** Basic characteristics of health care workers in study of proportion of needle stick injuries and their associated factors in tertiary care hospitals in Sindh province of Pakistan (n=497)

Characteristics	n (%)
Sex	
Male	178 (35.8)
Female	319 (64.2)
Area of practice	
Medicine	308 (62)
Surgery	189 (38)
Designation	
Doctor	235 (47.3)
Nurse	262 (52.7)
Years of practice	
< 5	350 (70.4)
> 5	147 (29.6)

Approximately 64% HCW (316/497) reported to have suffered from needle stick injuries. Amongst them, up to 27% workers (86/316) had it once, while nearly 73% (230/316) have suffered the injury twice or multiple times. Over two-third of HCW had not completed their vaccination against hepatitis B. Nearly two-fifths of HCW who suffered NSI were found to be involved in either injecting or drawing blood at the time of prick, while up to 37% have reported recapping of needle. Almost 45% of the workers considered long working hours to be the reason for NSI, while up to 42% considered inappropriate environment such as overcrowding to be the cause. Most frequently taken measure after sustaining the injury was found to be washing the injury with soap and water (20%), while up to 23% of the workers inquired about the patient's disease after being pricked (Table 2).

**Table 2:** Responses of health care workers to elements related to standard precautions in tertiary care hospitals in Sindh, Pakistan (n=497)

Characteristics	n* (%)
At least one needle stick injury	
Yes	316 (63.6)
No	181 (36.4)
Multiple needle stick injuries*	
< 2 times	86 (27.2)
≥ 2 times	230 (72.8)
Vaccinated for hepatitis B	
Yes	165 (33.2)
No	332 (66.8)
Activity during needle prick	
Injecting medicine/ drawing blood	208 (41.9)
Recapping of needle	182 (36.6)
Surgery or Suturing	106 (21.3)
Handling uncooperative patient	69 (13.9)
Reason of needle prick	
Long working hours	233 (46.9)
Inappropriate environment	218 (43.9)
Stress	60 (12.1)
Inappropriate training	50 (10.1)
Post exposure preventive measures	
Know about patients disease	111 (22.3)
Allow injury to bleed	99 (19.9)
Wash with soap, water and antiseptic	96 (19.3)
Notify infectious control person	7 (1.4)

* n – varies due to multiple responses

**Figure 1:**
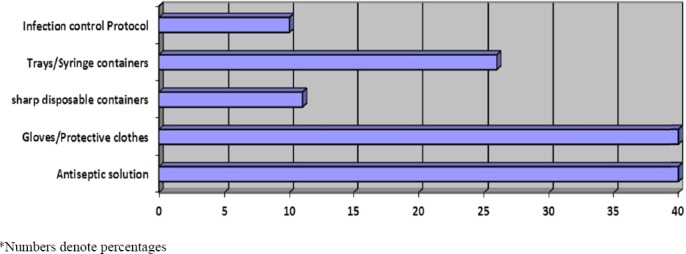
Availability of preventive facilities for needle sticks injuries for health care workers

Overall, only 40% of study participants reported to have availability of anti-septic solutions and gloves or protective cloths. About one-fourth HCWs reported to have trays/syringe containers while only 10% of the study participants had the availability of sharp/disposable containers and infection control guidelines/protocols in their working places (Figure 1).

In univariate analysis, odds of having NSIs were higher among HCWs who were: practicing for more than 5 years (OR=5.92, 95% CI: 3.45 – 10.16), nurses (OR=2.12, 95% CI: 1.35 - 3.32), working in surgical specialty (OR=1.6, 95% CI: 1.09 - 2.51) and females (OR=1.52, CI: 1.04-2.22) and had received booster doses for hepatitis B vaccine (OR=1.85, 95% CI: 1.10 – 3.11) (Table 3).

**Table 3:** Univariate analysis for factors associated with needle stick injuries (NSIs) among health care workers in tertiary care hospitals in Sindh, Pakistan (n=497)

Characteristics	All (%)	Had NSI n (%)	p-value^1^	OR (95% CI)
Years of practice			<0.001	
< 5	350 (70.4)	188 (53.7)		1.0
> 5	147 (29.6)	128 (87.1)		5.92 (3.45-10.16)
Designation			0.001	
Doctor	235 (47.3)	131 (55.7)		1.0
Nurse	262 (52.7)	185 (70.6)		2.12 (1.35-3.32)
Received booster dose of hepatitis B vaccine			0.02	
Yes	109 (22)	77 (70.6)		1.0
No	387 (78)	238 (61.5)		1.85 (1.10-3.11)
Area of practice			0.01	
Medicine	308 (62)	184 (59.7)		1.0
Surgery	189 (38)	132 (69.8)		1.6 (1.09-2.51)
Sex			0.83	
Males	178 (35.8)	102 (57.3)		1.0
Females	319 (64.2)	214 (67.1)		1.52 (1.04-2.22)

**p-value^1^:** of chi-squared test

## 4. Discussion

Our study estimated that the prevalence of NSIs is high among HCWs in Sindh, Pakistan. Factors found to be associated with this high prevalence were being female, nurse and working in surgical department. In addition, working for >5 years and having received booster dose of hepatitis B vaccination were also associated with NSIs. Furthermore, it highlights the inadequate adherence to the universal precautionary measures. Such high occurrence of NSIs predisposes HCWs to pathogens transmitted through blood (Pruss-Ustun et al., 2005; WHO, 2002; World health report, Geneva). Hence, efforts to decrease NSIs should focus on above key target areas which may lead to a substantial reduction in the burden of blood transmitted diseases like hepatitis B and C which are on the rise in Pakistan and are relatively higher in HCWs than in general population (Ali, Donahue, Qureshi, & Vermund, 2009).

Although our results are similar to the studies of HCWs from hospitals of Alexandria (Hanafi, Mohamed, Kassem, & Shawki, 2011) and Turkey (Azap et al., 2005); the prevalence of NSIs reported in our study is considerably higher than those reported from two previous studies from our own country, Pakistan (Zafar et al., 2008; Zafar et al., 2009). The low prevalence of NSI, reported in earlier studies, could be due to the hospital being private; the existence of infection control program; inclusion of infection control as a subject in curriculum of nursing staff; regular educational activities; surveillance and use of gloves in the study hospital. It may also be result of underreporting by HCWs. Perhaps it reflects the true situation, however, this could be explained by Hawthorne effect (French Jr, 1950) which implies the improvement in behavior of participants if they know that they are being observed. Interestingly, our study findings are in contrast with that of another study from Pakistan (Siddique et al., 2008) which reported a very high rate (94%) of NSIs among HCWs. This can be attributed to the ‘selection bias’ as majority of participants were from surgical departments and emergency room and studies have reported highest risk of NSIs in these areas (Äİlhan, Durukan, Aras, Türkçüoğlu, & Aygün, 2006; Nsubuga & Jaakkola, 2005).

Our results are consistent with the findings from different studies that female HCWs (Hanafi et al., 2011), particularly nurses (Ghannad, Majzoobi, Ghavimi, & Mirzaei, 2012; Gillen et al., 2003; Muralidhar, Singh, Jain, Malhotra, & Bala, 2010; Rampal, Zakaria, Sook, & Zain, 2010) sustained the highest NSIs as compared to other HCWs. Females constitute bulk of nursing profession in developed as well as developing countries (Zurn, Dal Poz, Stilwell, & Adams, 2004), injections are mainly administered by nurses and also they are often involved in drawing of blood; thus they are most exposed and vulnerable to NSIs than other HCWs.

Generally studies report that long work experience is associated with decreased risk of NSI among HCWs (Hanafi et al., 2011; Rampal et al., 2010) and a study from Malaysia reports no significant difference in prevalence of NSI based on duration of work experience; however our findings are in contrast with these results. This could be explained by more exposure due to longer duration of services and hence more NSI among more experienced HCWs as compare to those working for < 5 years. This could also be a reflection of poor injection practices and training of HCWs with long job history. It implies proper training of HCWs, particularly senior professionals, with refreshers.

We found that long working hours results in increase in NSI; this is in congruence with the findings from studies from Pakistan (Aslam et al., 2010; Zafar et al., 2008) and other countries (Äİlhan et al., 2006; Olds & Clarke, 2010). This may be a result of mental and physical stress (Nsubuga & Jaakkola, 2005) associated with excessive working hours. It also suggests the problem of understaffing in developing countries (Awases, Gbary, Nyoni, & Chatora, 2004). Hence it has implications for policy makers and hospital administrators to ensure that working hours do not exceed than those prescribed in legislation.

Two thirds of our study participants were not completely vaccinated against hepatitis B virus (HBV). These findings are very low in comparison to the study from developed countries like USA and UK (Gershon et al., 2005; Puro et al., 2005). This may reflect poor accessibility and affordability in developing countries. Nevertheless, keeping in view the high prevalence of hepatitis B and C in HCWs compared to general population of Pakistan (Ali et al., 2009), this dismal vaccine coverage puts HCWs at high risk of infection and warrants immediate attention by policy makers. Interestingly, rate of NSIs was high among those HCWs who have received booster dose of hepatitis B vaccine. This paradox could be explained by false sense of protection among them which may lead to careless behavior and subsequent NSIs. However, further exploration is required in this context.

Adherence to pre and post exposure precautions is essential to decrease provider to patient (and vice versa) transmission of blood borne pathogens. Substantial number (42%) of HCWs got NSIs while injecting or drawing blood, this estimate is lower than earlier reported of 53% by another study from Pakistan (Zafar et al., 2008). Although, this may indicate improved practice of HCWs, however there is still room for improvement. It implies further training of HCWs in such procedures. Needle recapping was found as the second most common procedure that resulted in 37% of NSIs. This finding is consistent with that of other studies from Pakistan (Aslam et al., 2010; Zafar et al., 2008). Besides poor clinical practices, this could be due to very limited availability of sharp disposable containers in hospitals as reported by only 10% of HCWs in this study. Similarly, very low statistics were reported for availability of infection control guidelines and use of gloves. This finding is in line with a study from Pakistan (Aslam et al., 2010) that also reported very low use of gloves and other protective equipment. Nevertheless, this situation deserves immediate attention by hospital administrators and health policy makers that can potentially reduce the risk of transmission of blood borne pathogens from patients to HCWs and vice versa.

Results from this study should be interpreted with caution as this is a cross sectional survey and we included only tertiary care hospitals from urban areas so results cannot be generalized to the HCWs of rural hospitals and/or first level care facilities which might have lesser training opportunities for their HCWs, and inappropriate availability of preventive facilities.

## 5. Conclusion

This study accentuates very high levels of NSI along with very low vaccination coverage, poor safety practices and inadequate preventive facilities. Formal training and provision of preventive facilities should be ensured by health authorities in the study hospitals.
